# Supplementary material for: The Structure–Property Relationship of Pyrrolidinium and Piperidinium-Based Bromide Organic Materials
Source: Materials (Basel). 2022 Nov 28;15(23):8483. doi: 10.3390/ma15238483 (PMC9737136; doi:10.3390/ma15238483)
Supplement: Supplementary file 1 [file materials-15-08483-s001.zip › materials-2008119-supplementary.pdf]

Structure-property relationship study of pyrrolidinium and piperidinium-based bromide organic materials

Claudio Ferdeghini[1], Andrea Mezzetta[1], Felicia D'Andrea[1], Christian Silvio Pomelli[1], Lorenzo Guazzelli[1], and Luca Guglielmero[1,2*]

[1] Department of Pharmacy, University of Pisa, Via Bonanno 33, Pisa 56126, Italy;

[2] Scuola Normale Superiore, Piazza dei Cavalieri 7, 56126, Pisa, Italy.

* luca.guglielmero@sns.it

Supporting Information

Table of Contents

NMR spectra of compounds **1-4**	pages S2-S7
TGA profiles of compounds **1-4**	pages S8-S9
DSC thermograms of compounds **1-4**	pages S10-S12
ESI-MS spectra of compounds **1-4**	pages S13-S14
ATR-FTIR spectra of compounds **1-4**	pages S15-S16
Kofler determination of [C_3Mpyrr$_2$]2Br (**1**) melting point	page S17

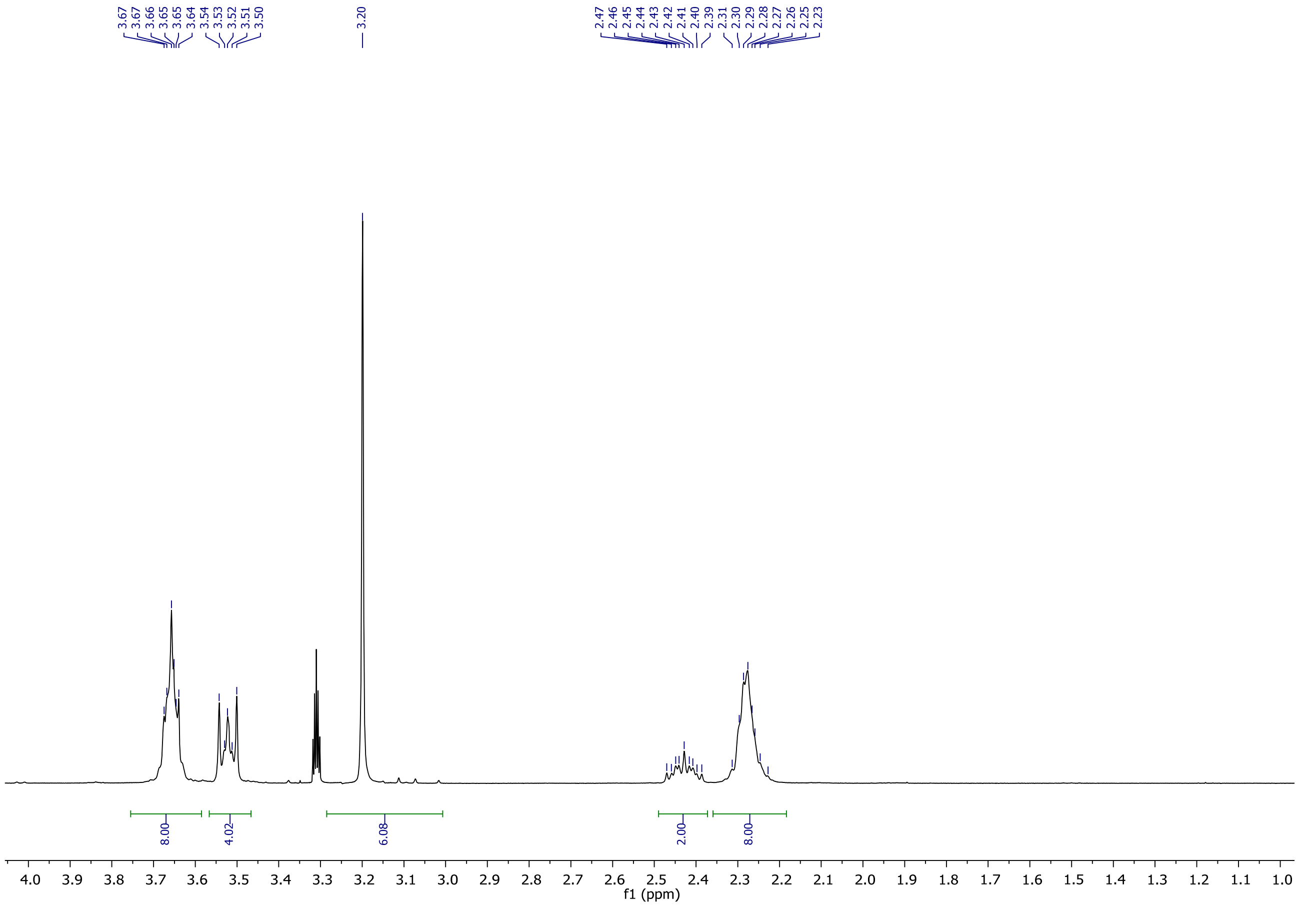

Figure S1. ^{1}H-NMR spectrum of [C_3Mpyrr$_2$]2Br (**1**)

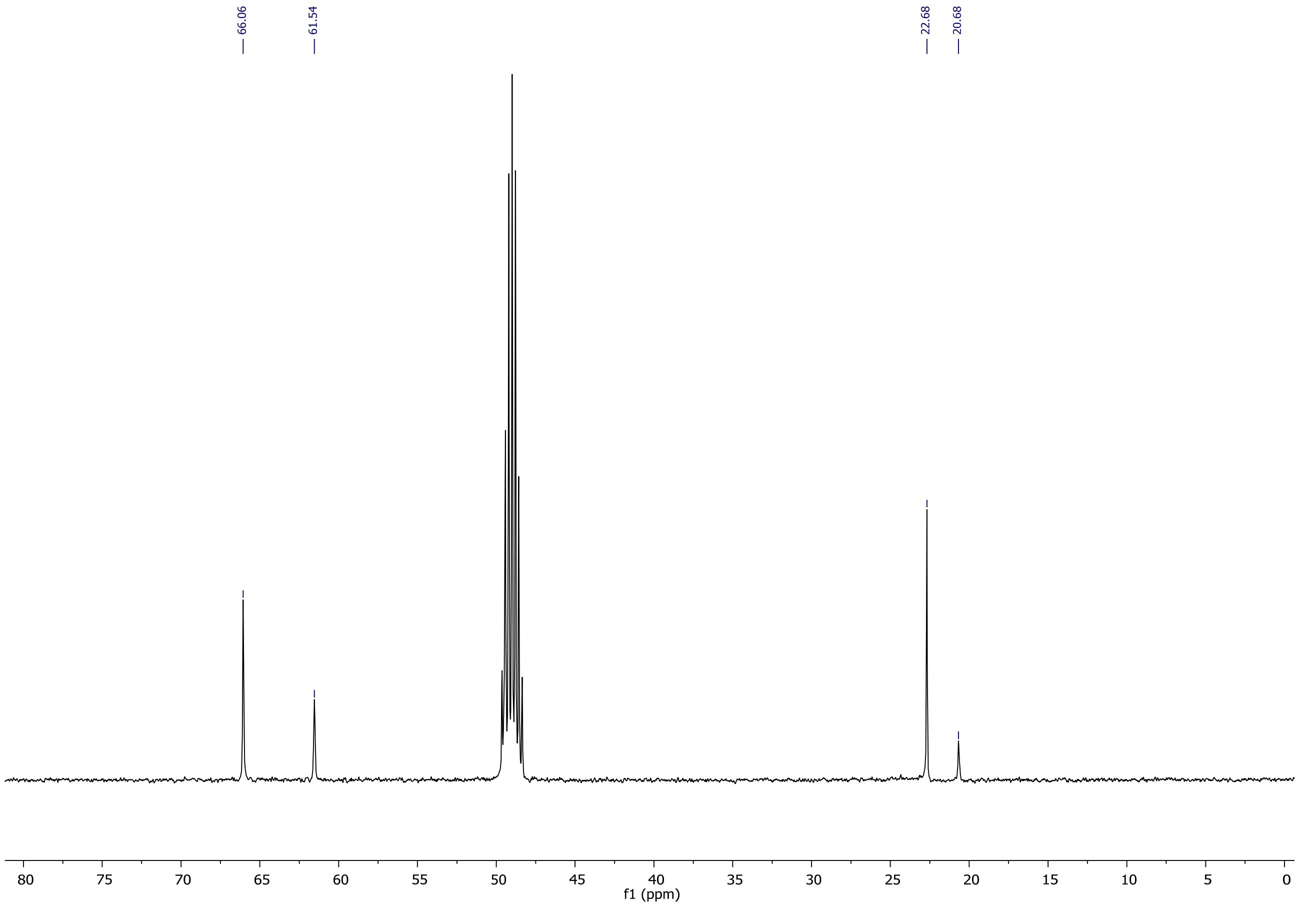

Figure S2. ^{13}C-NMR spectrum of [C_3Mpyrr$_2$]2Br (**1**)

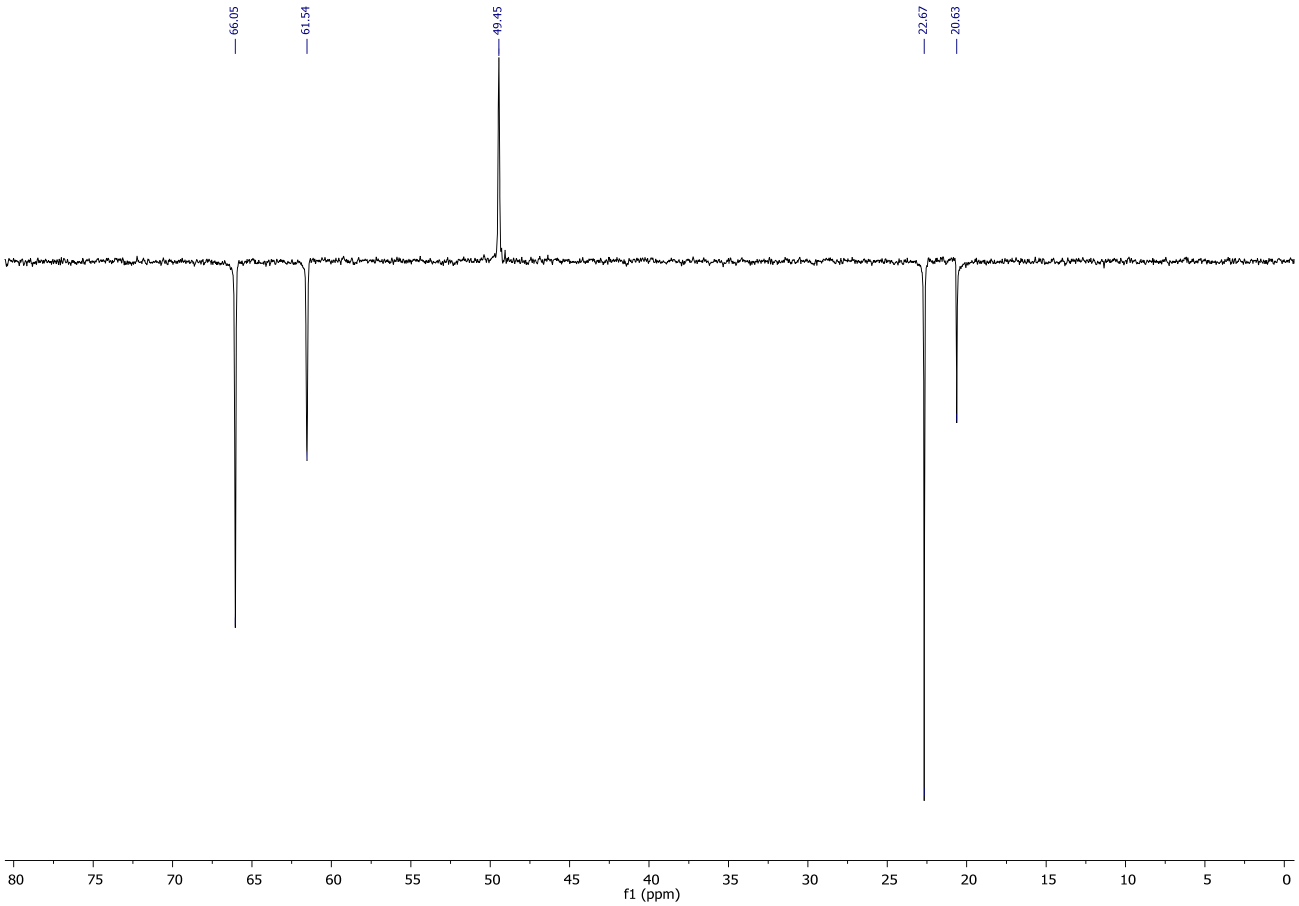

Figure S3. DEPT-135 NMR spectrum of [C_3Mpyrr$_2$]2Br (**1**)

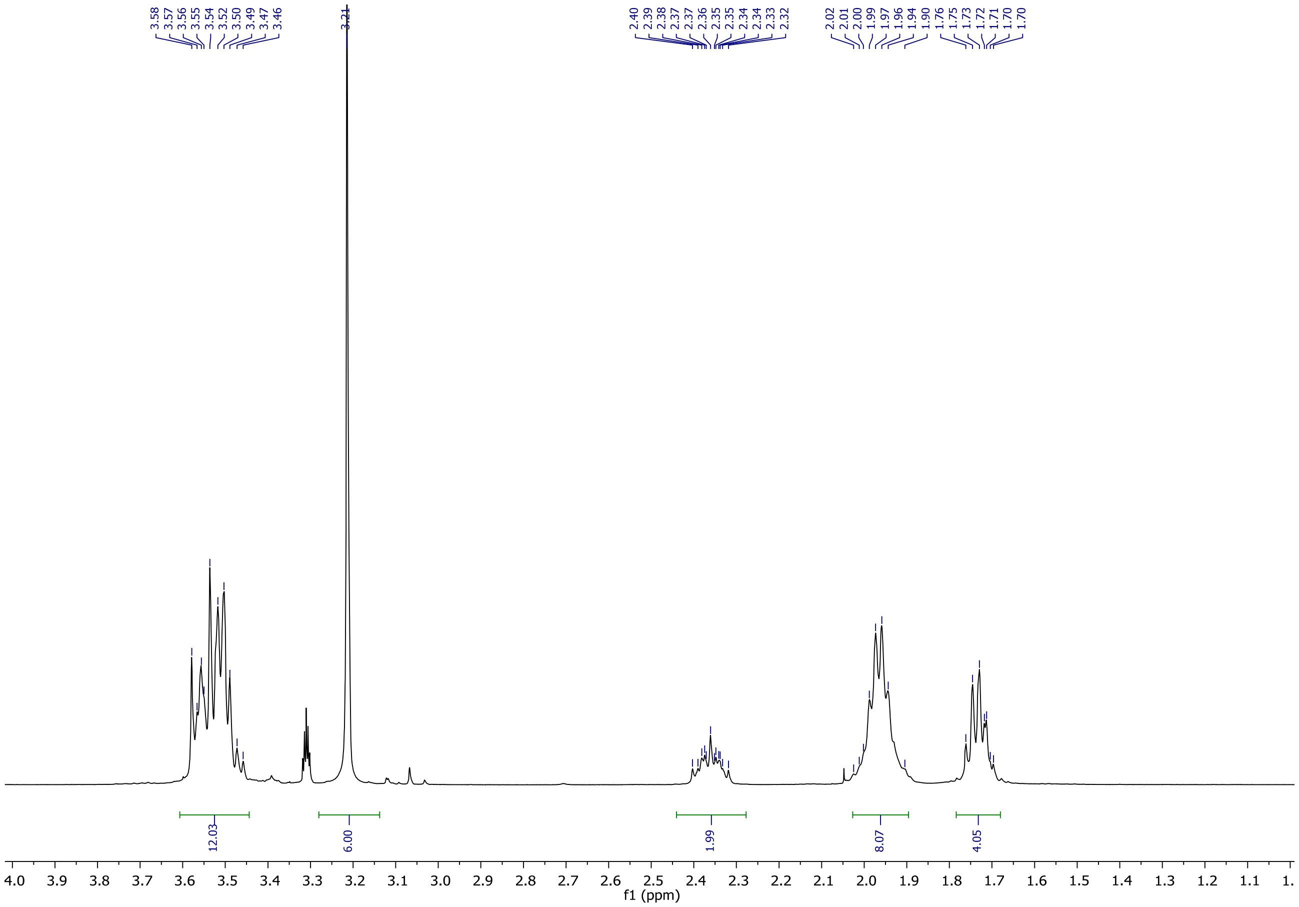

Figure S4. ^{1}H-NMR spectrum of $[C_3Mpip_2]2Br$ (**2**)

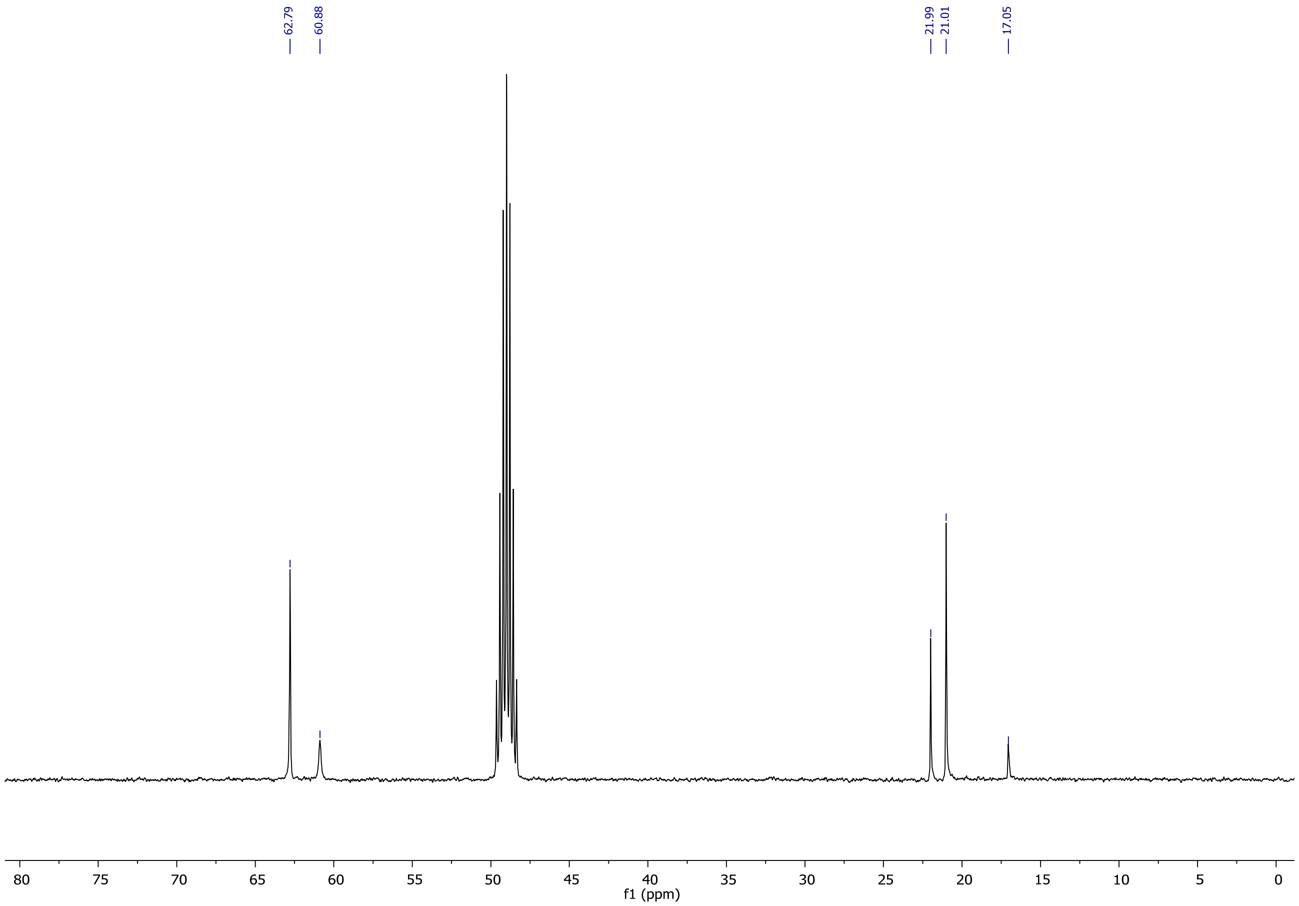

Figure S5. ^{13}C-NMR spectrum of $[C_3Mpip_2]2Br$ (**2**)

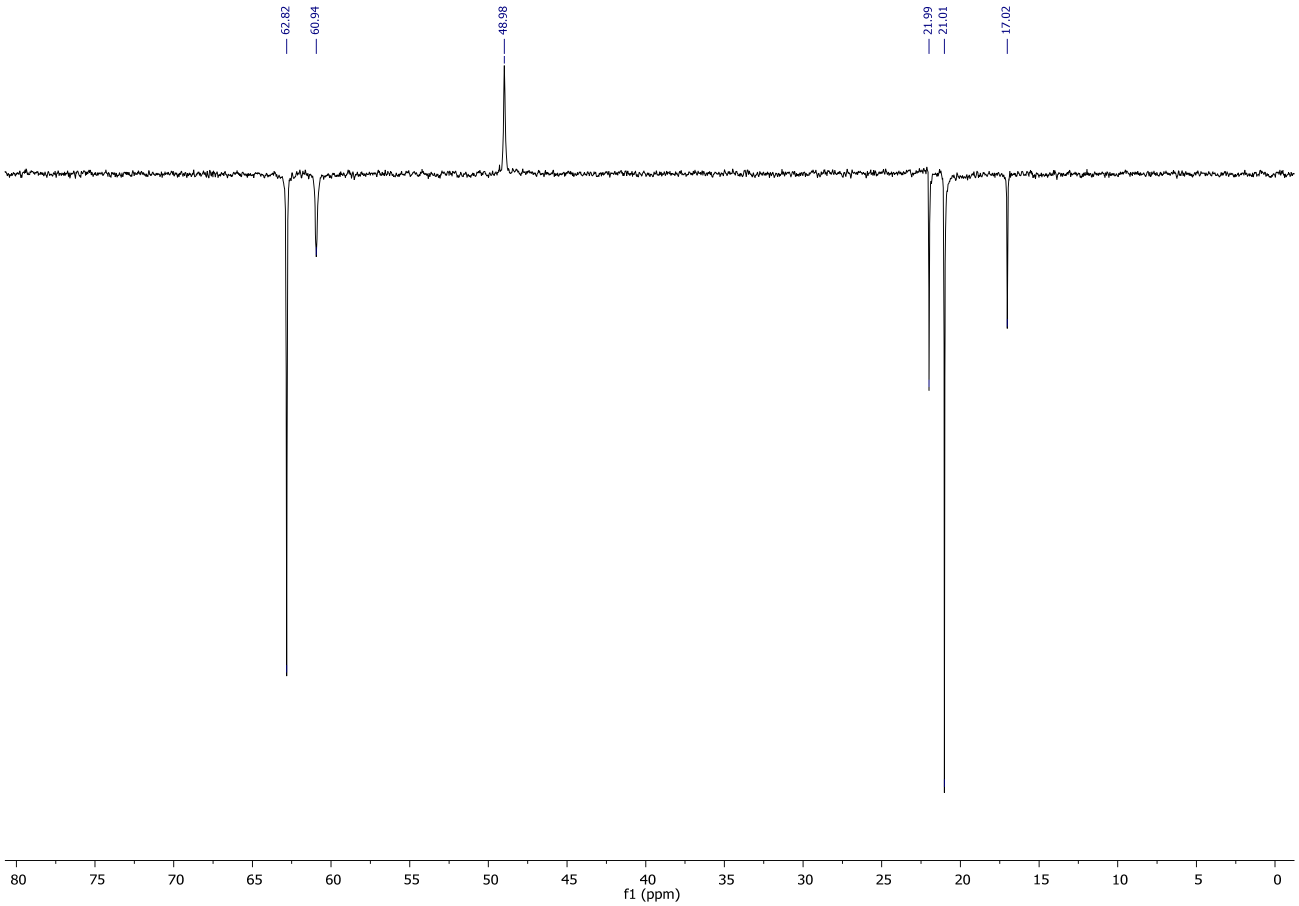

Figure S6. DEPT-135 NMR spectrum of [C_3Mpip_2]2Br (**2**)

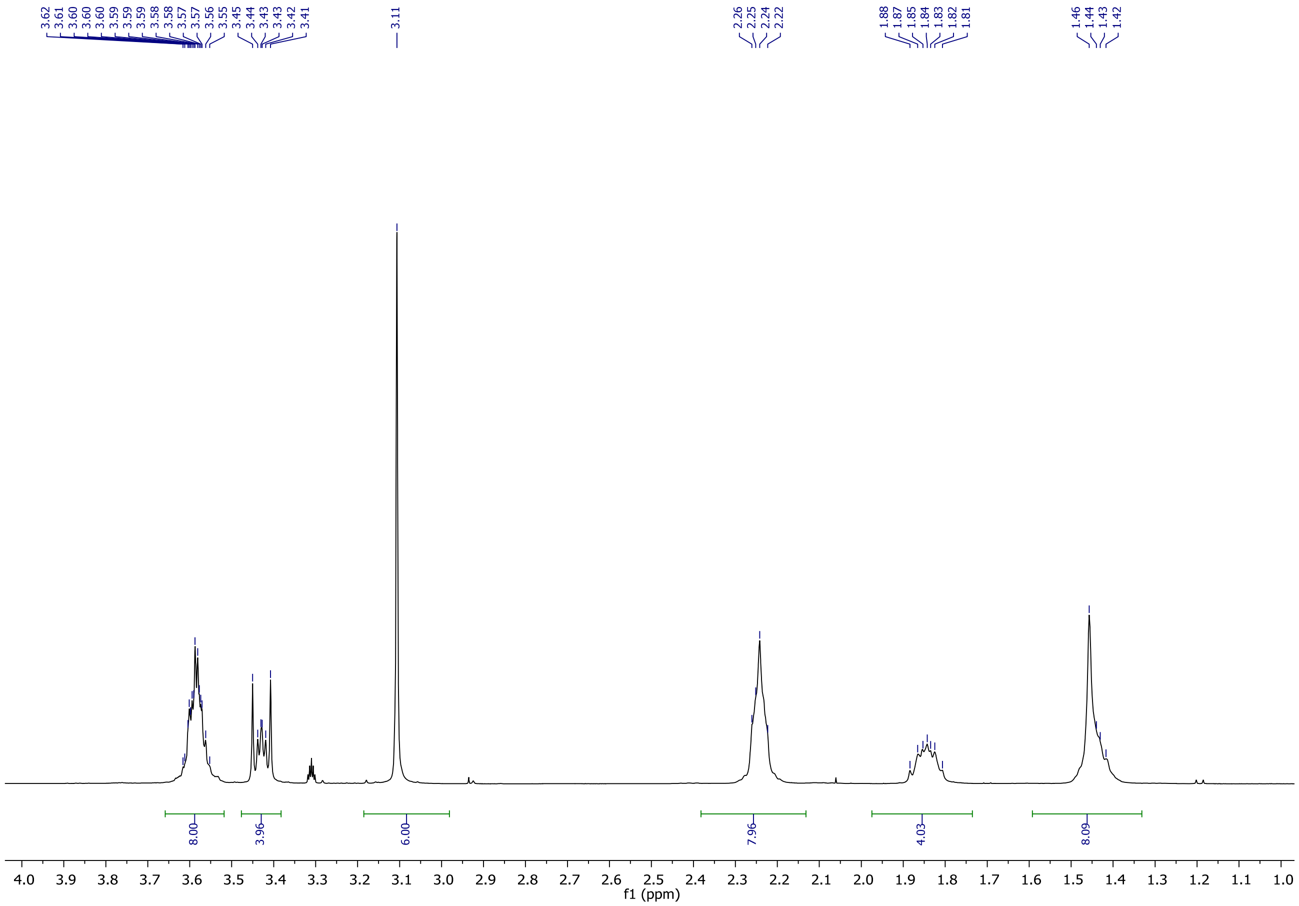

Figure S7. ^{1}H-NMR spectrum of [C_8Mpyrr$_2$]2Br (**3**)

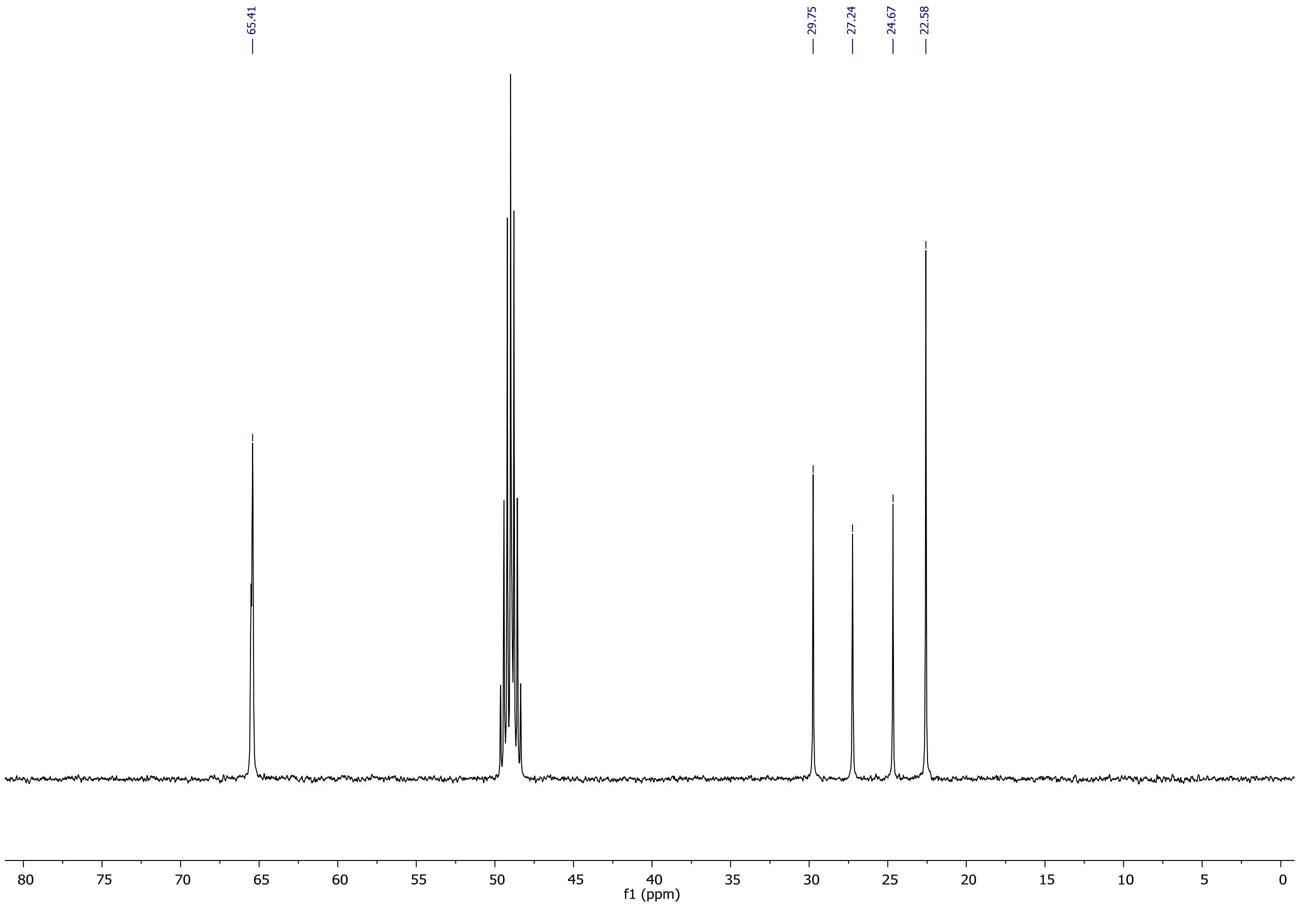

Figure S8. ^{13}C-NMR spectrum of [C_8Mpyrr$_2$]2Br (**3**)

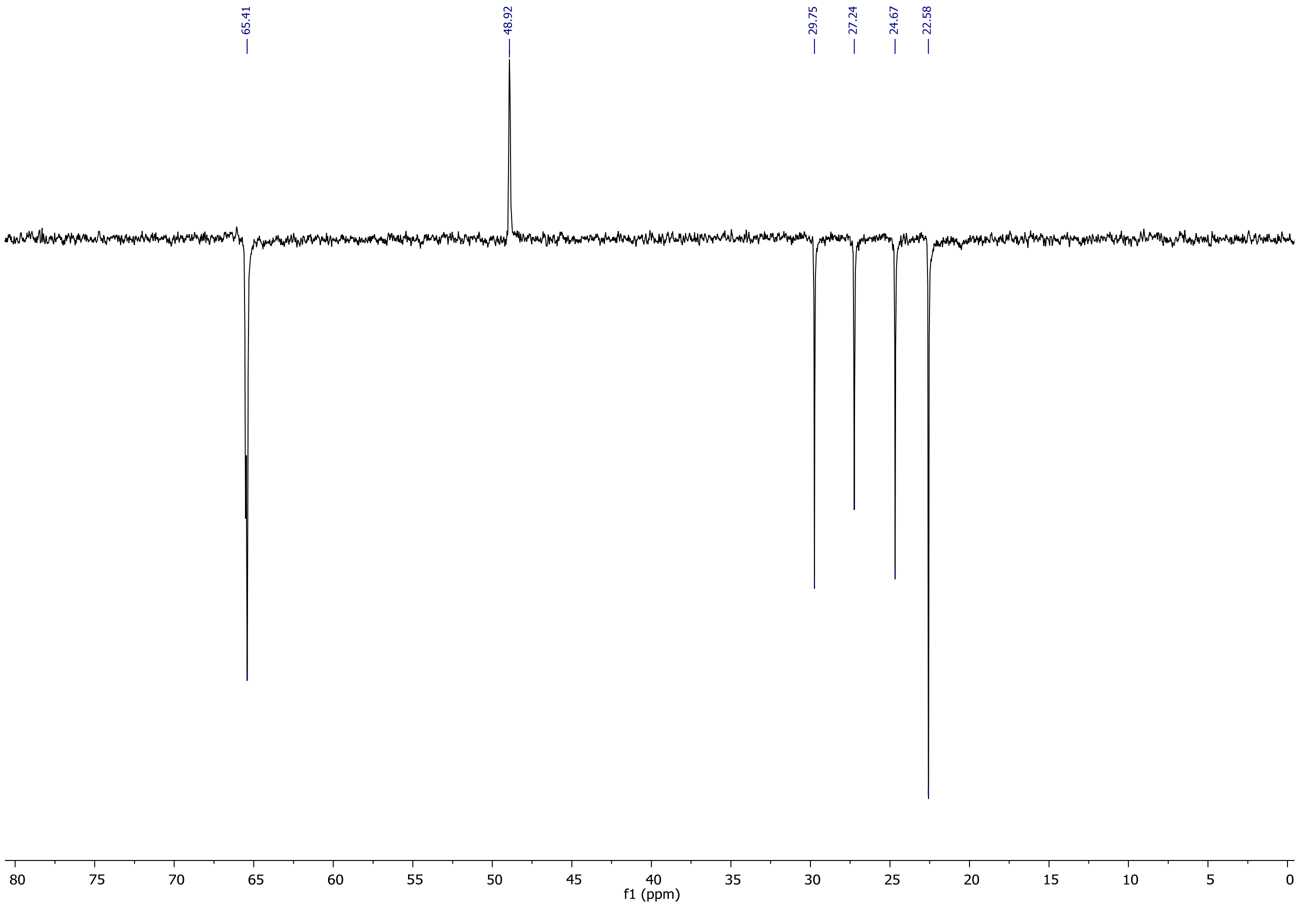

Figure S9. DEPT-135 NMR spectrum of [C_8Mpyrr$_2$]2Br (**3**)

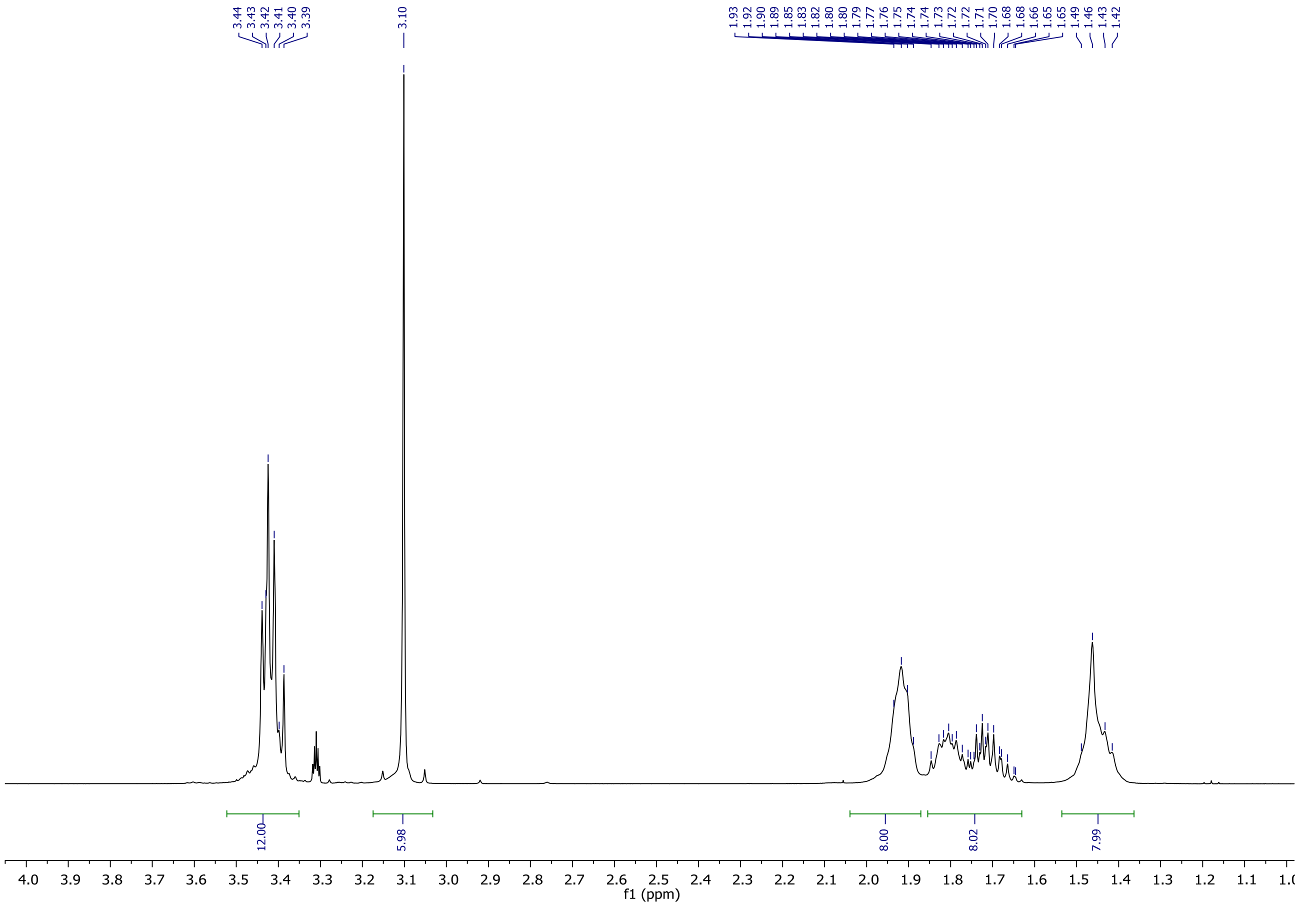

Figure S10. ^{1}H-NMR spectrum of $[C_8Mpip_2]2Br$ (**4**)

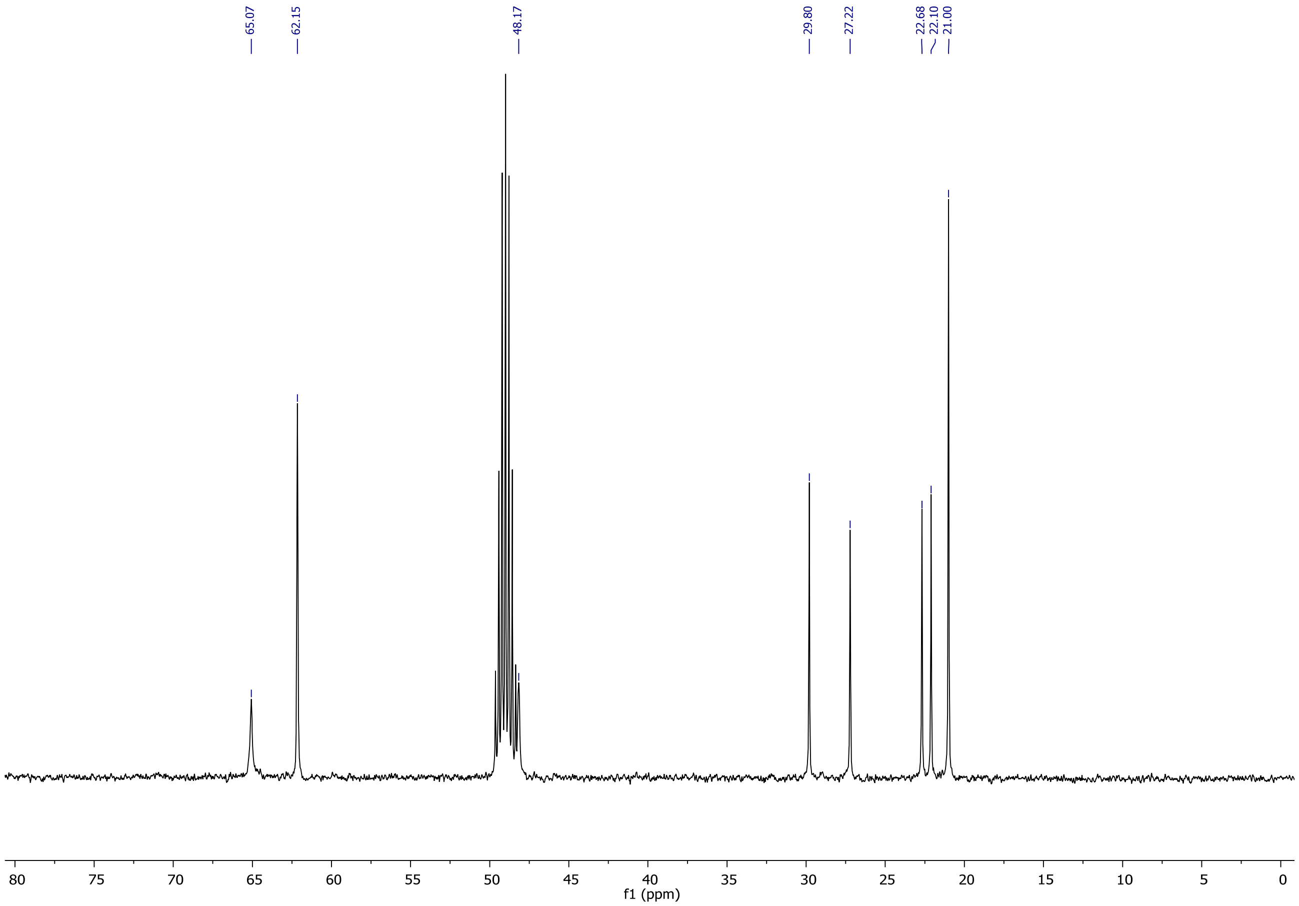

Figure S11. ^{13}C-NMR spectrum of [C_8Mpip$_2$]2Br (**4**)

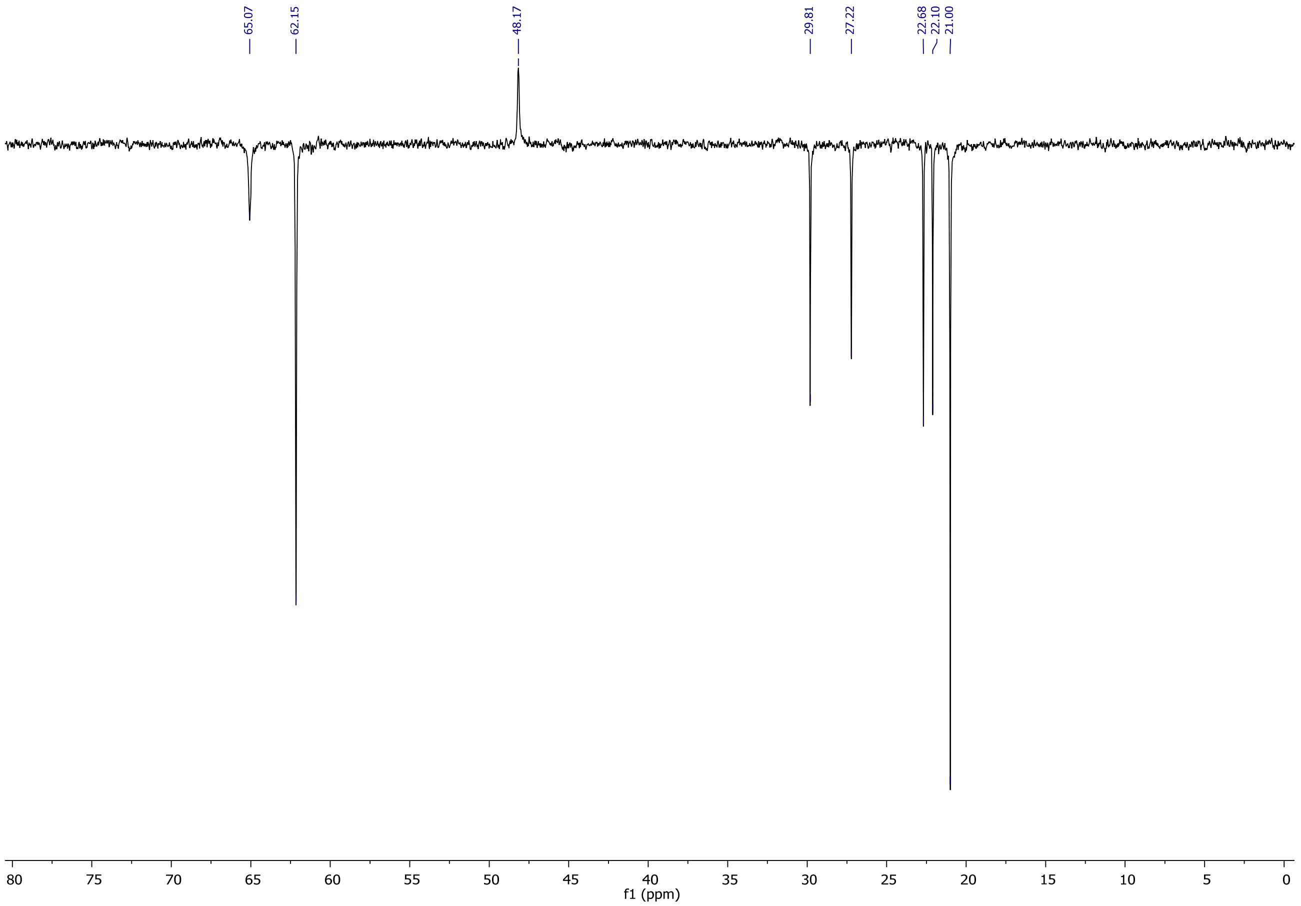

Figure S12. DEPT-135 NMR spectrum of [C_8Mpip$_2$]2Br (**4**)

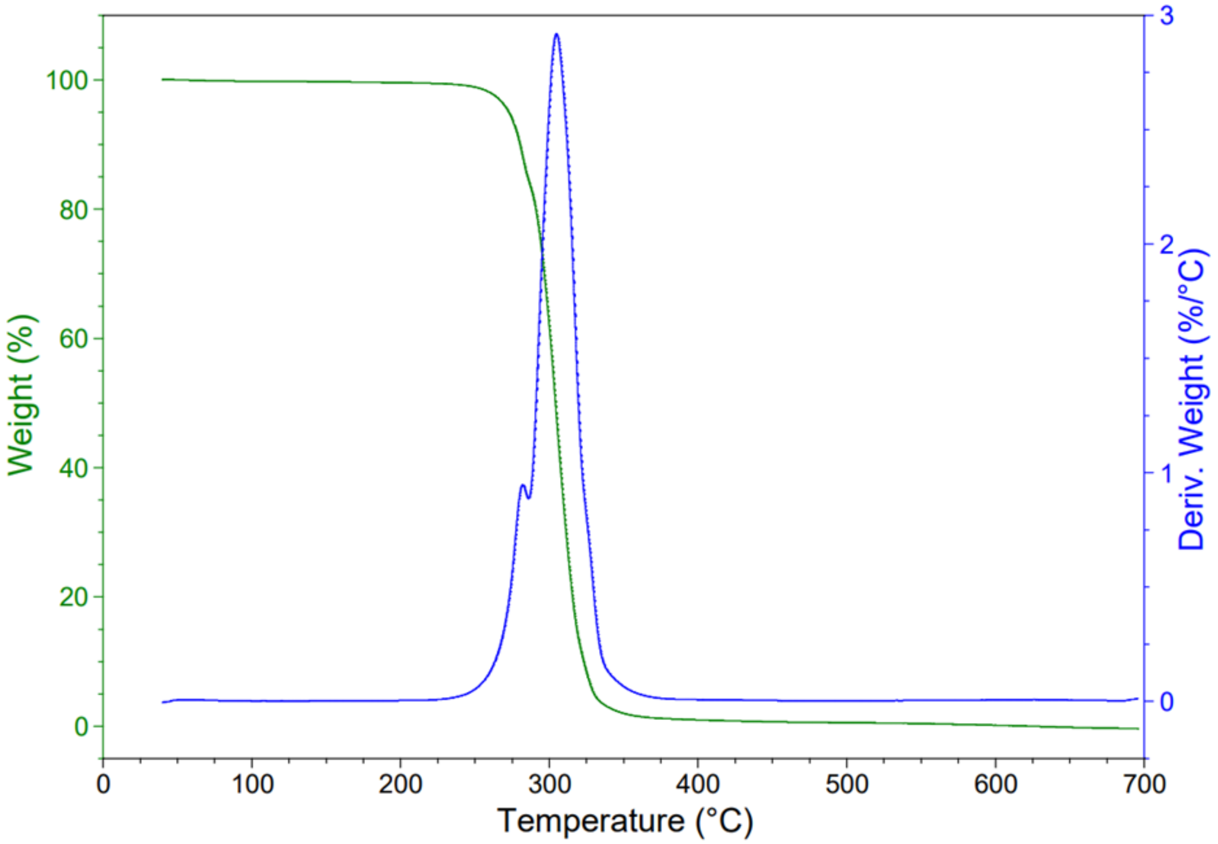

Figure S13. Thermal gravimetric analysis and derivative of [C_3Mpyrr$_2$]2Br (**1**)

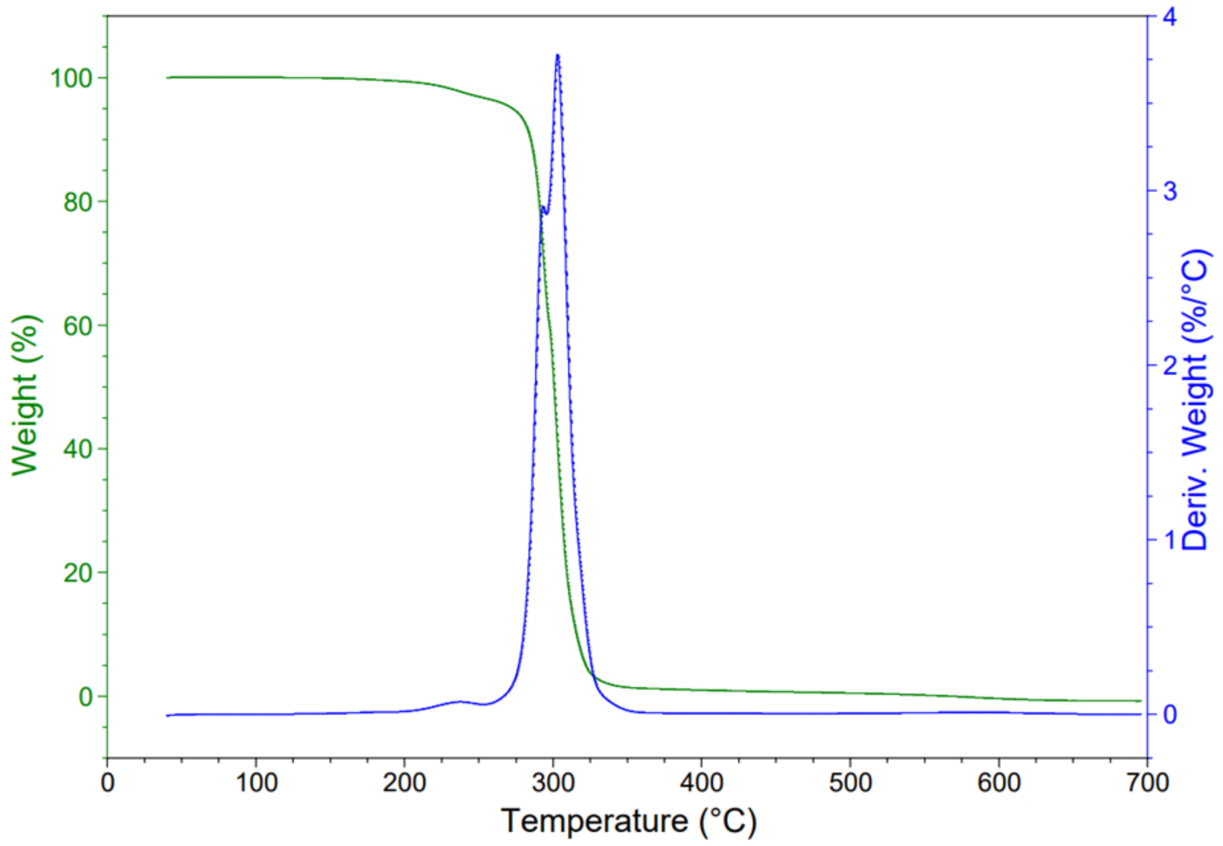

Figure S14. Thermal gravimetric analysis and derivative of [C_3Mpip$_2$]2Br (**2**)

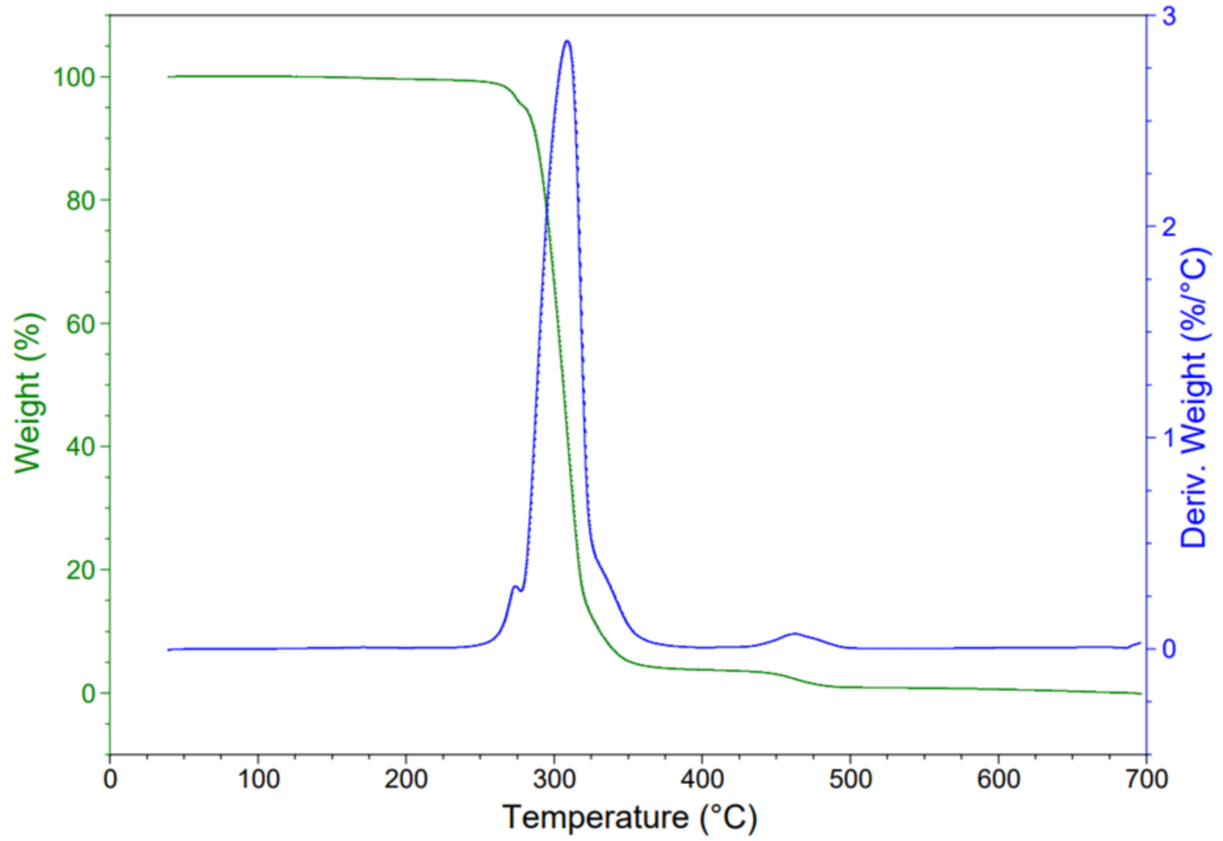

Figure S15. Thermal gravimetric analysis and derivative of [C_8Mpyrr$_2$]2Br (**3**)

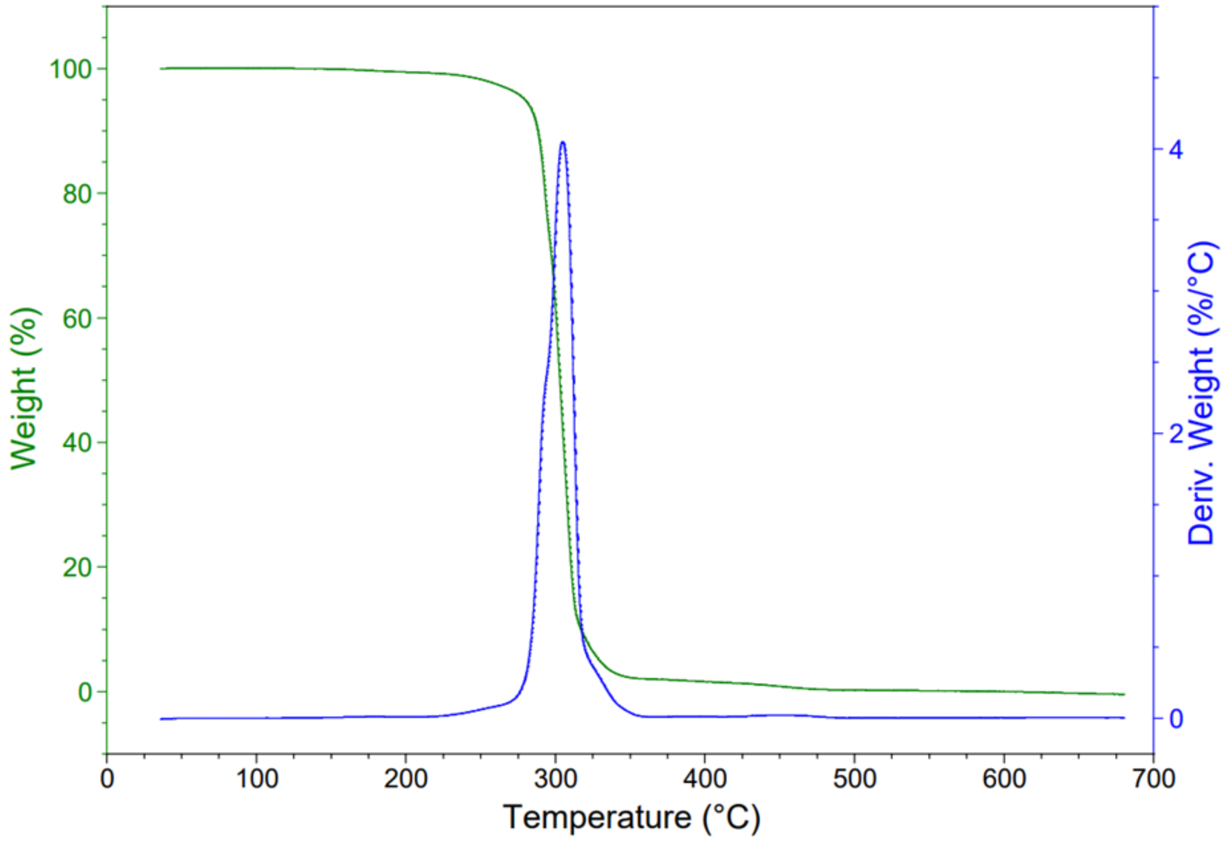

Figure S16. Thermal gravimetric analysis and derivative of [C_8Mpip$_2$]2Br (**4**)

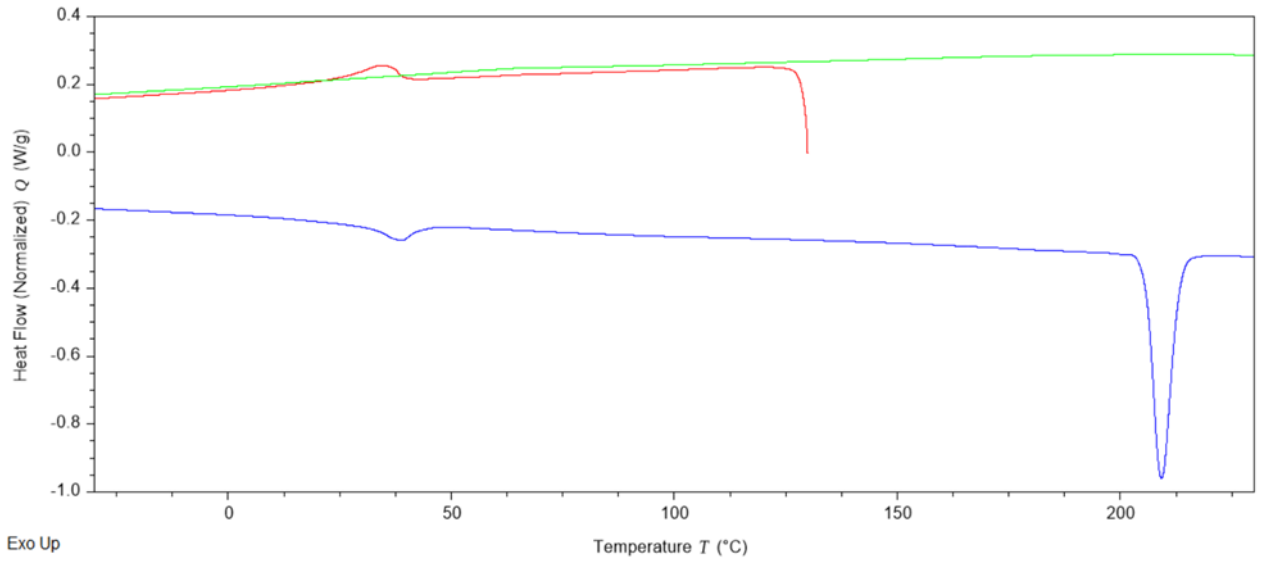

Figure S17. DSC analysis of [C_3Mpyrr$_2$]2Br (**1**).

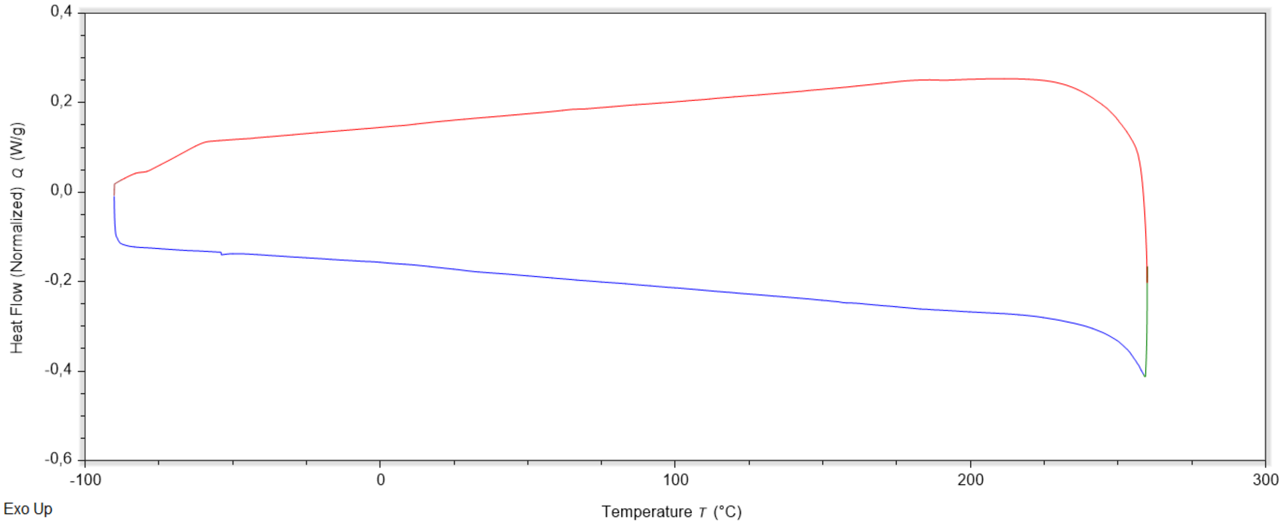

Figure S18. DSC analysis of [C_3Mpip$_2$]2Br (**2**)

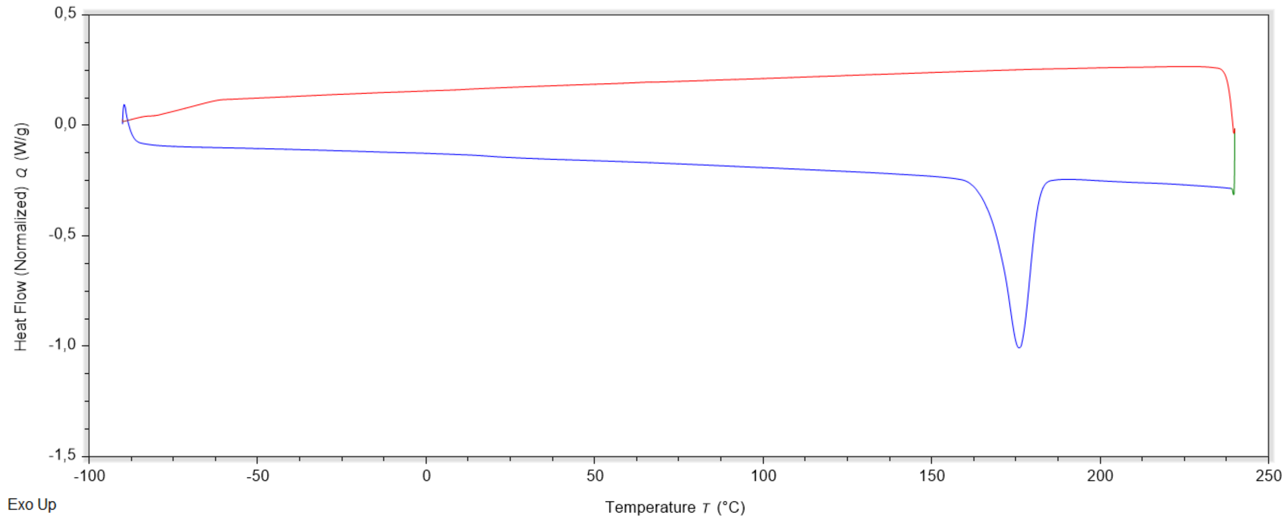

Figure S19. DSC analysis of [C_8Mpyrr$_2$]2Br (**3**)

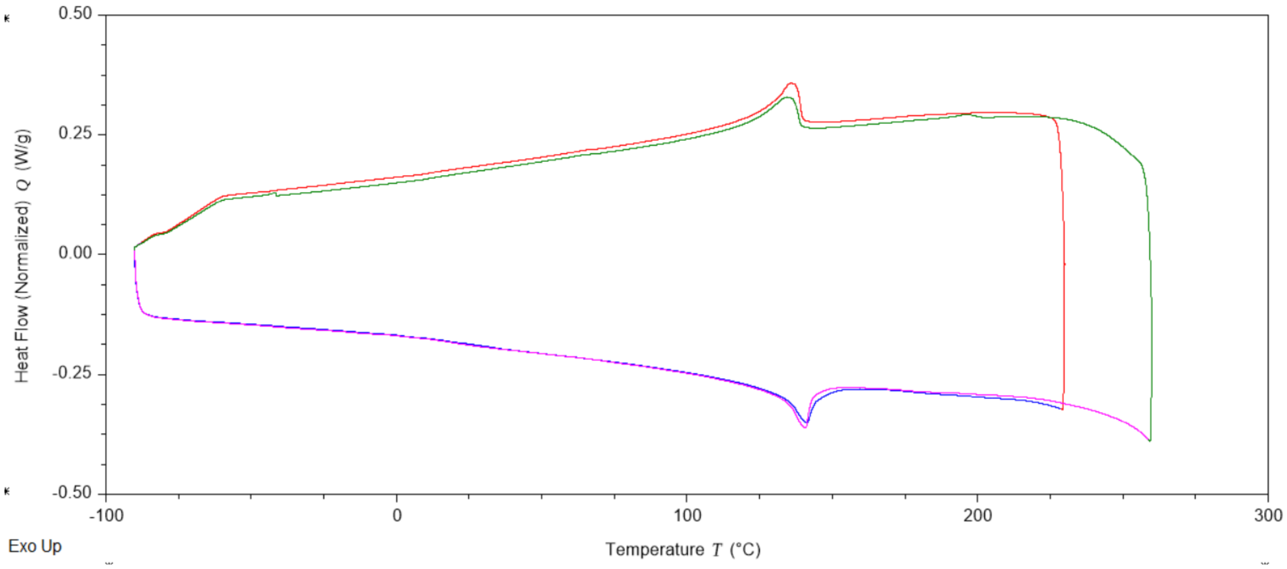

Figure S20. DSC analysis of [C_8Mpip$_2$]2Br (**4**)

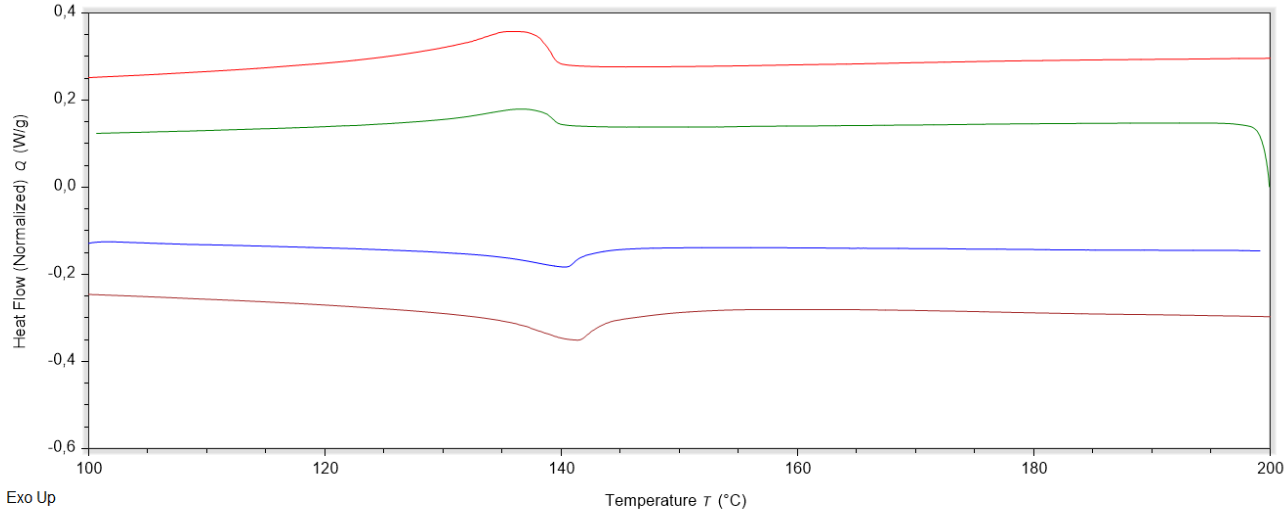

Figure S21. DSC analysis of $[C_8Mpip_2]2Br$ (**4**): overlap of the runs at 10 and 5 °C min^{-1}

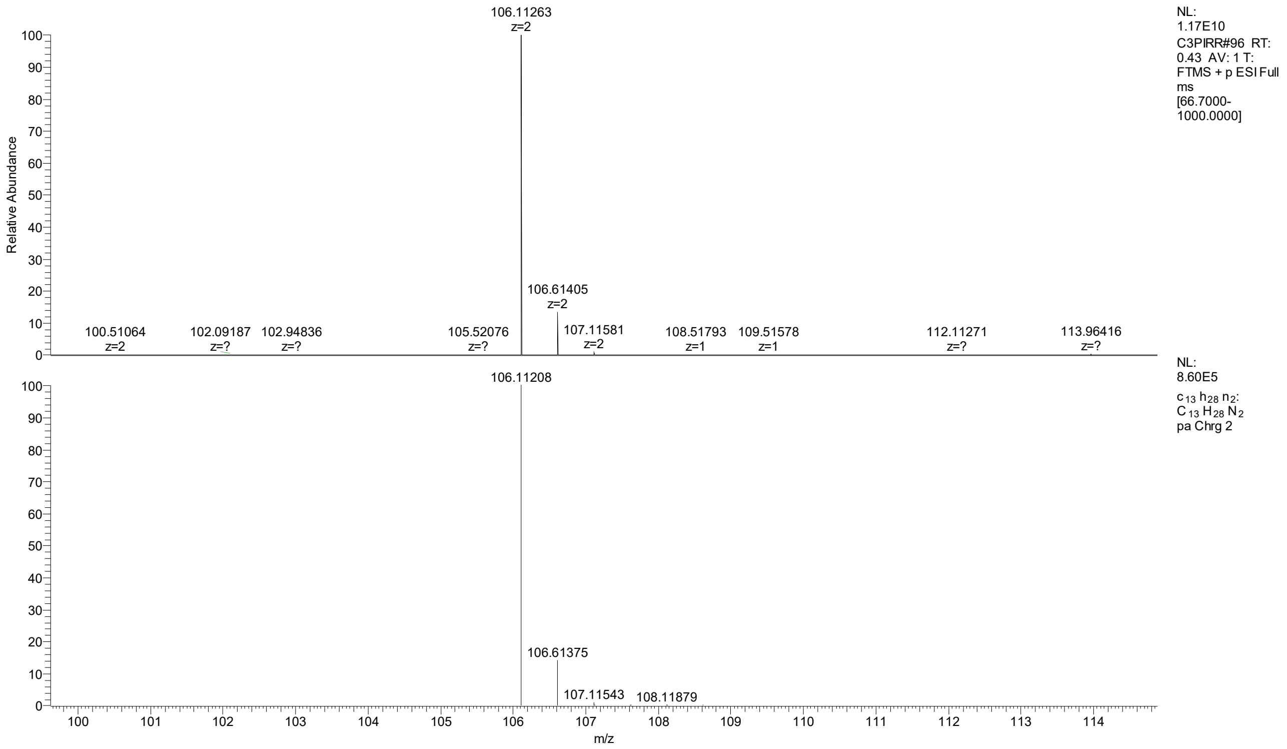

Figure S22. ESI-MS (positive mode) spectra of [C_3Mpyrr$_2$]2Br (**1**) calculated (below) and found (above).

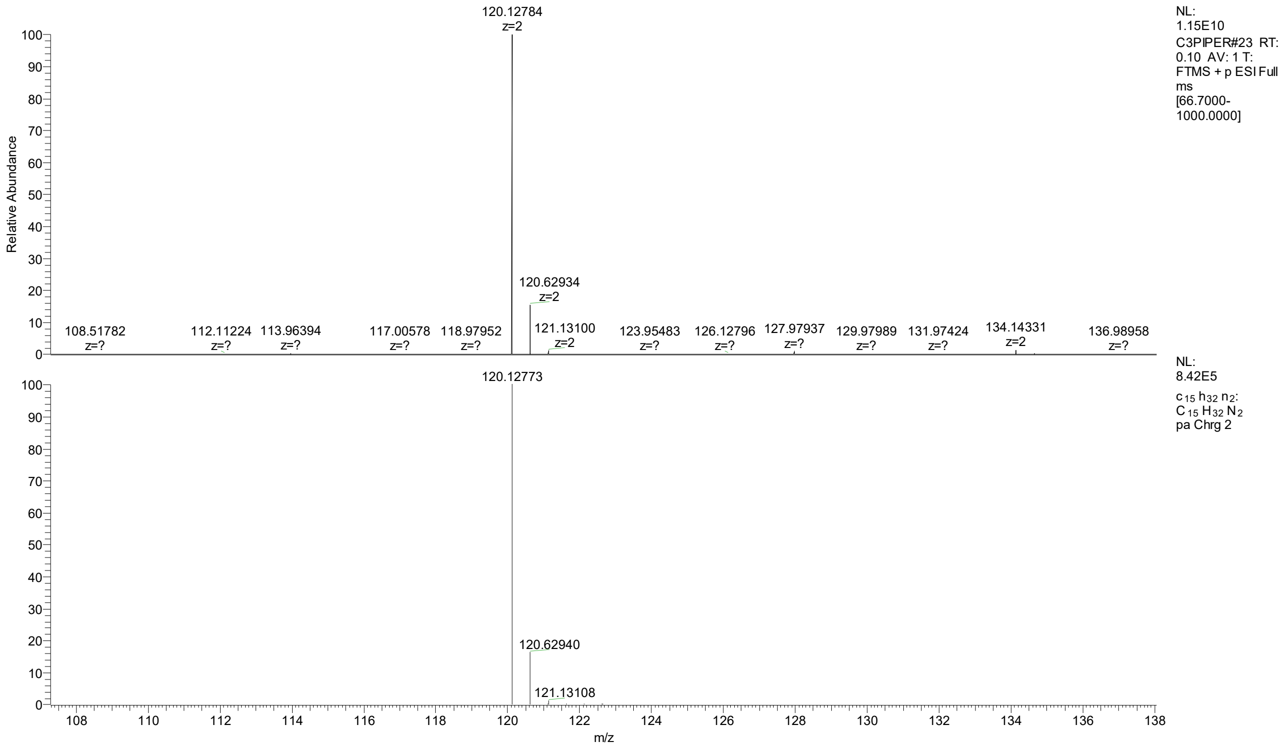

Figure S23. ESI-MS (positive mode) spectra of [C_3Mpip$_2$]2Br (**2**) calculated (below) and found (above).

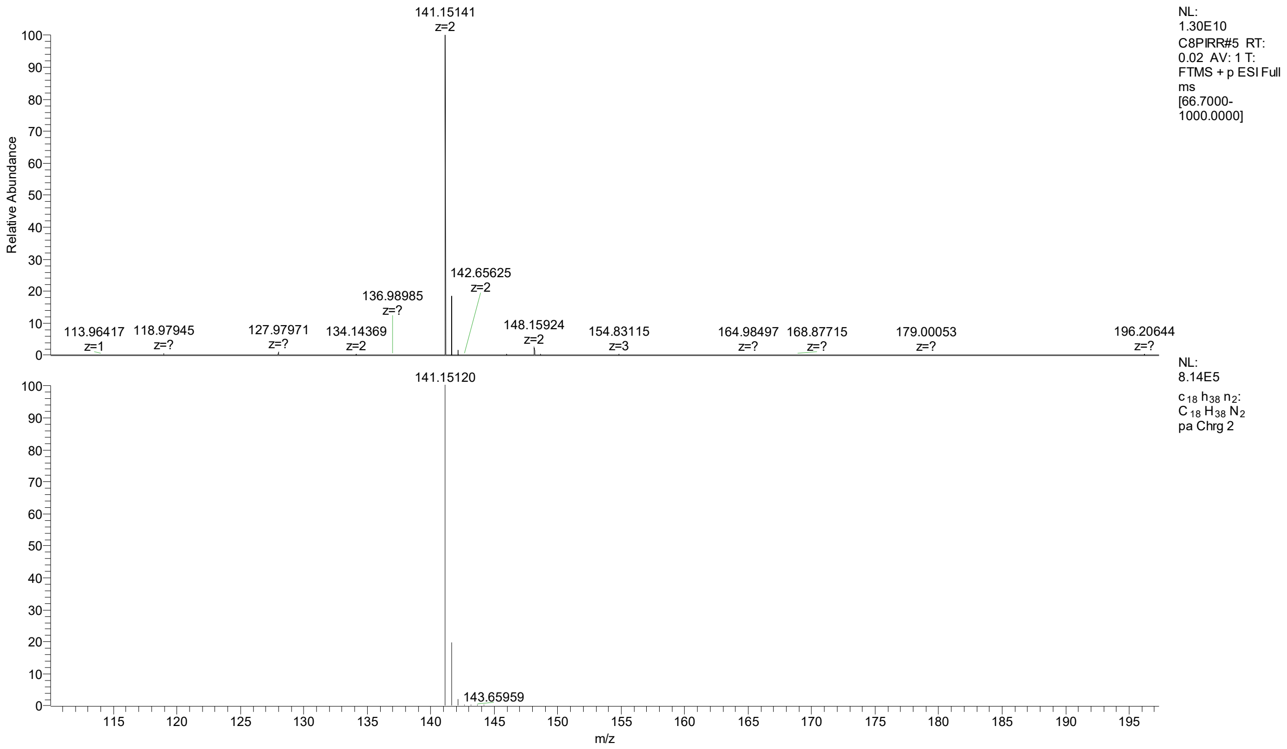

Figure S24. ESI-MS (positive mode) spectra of [C_8Mpyrr$_2$]2Br (**3**) calculated (below) and found (above).

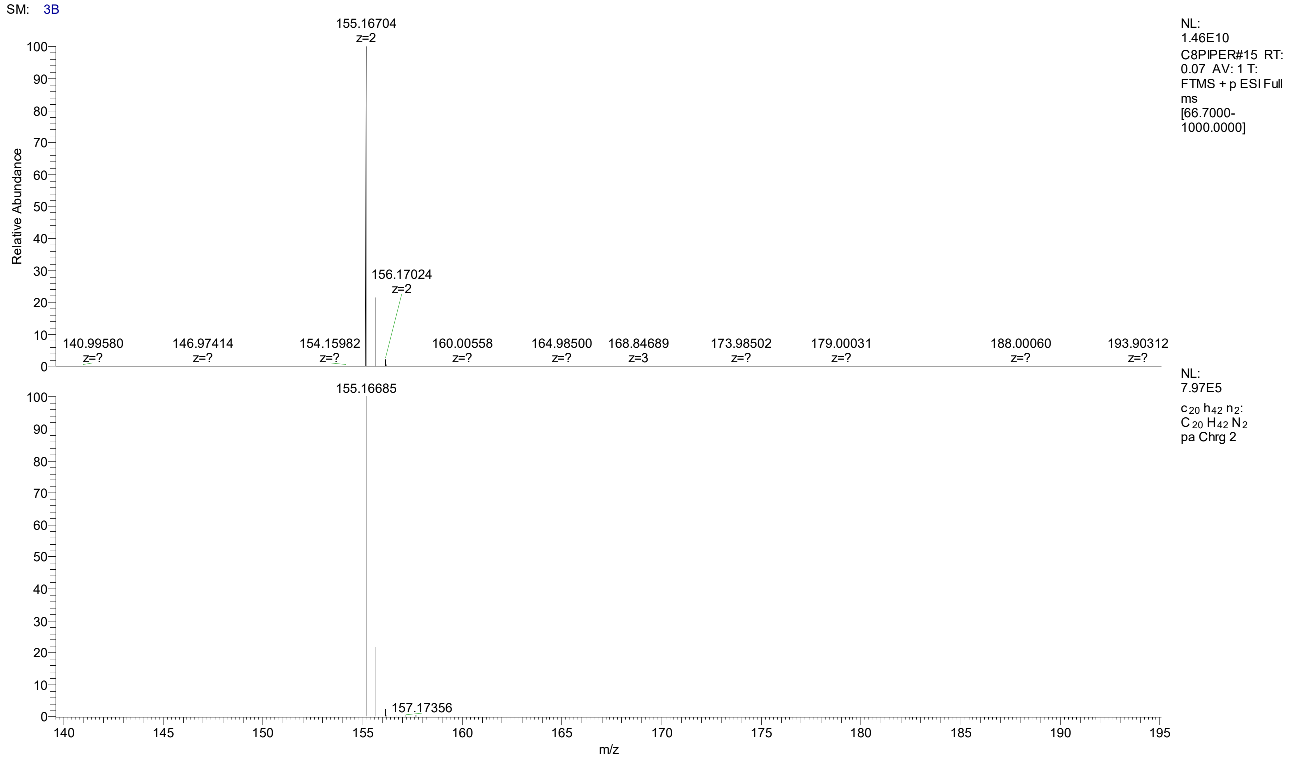

Figure S25. ESI-MS (positive mode) spectra of [C_8Mpip$_2$]2Br (**4**) calculated (below) and found (above).

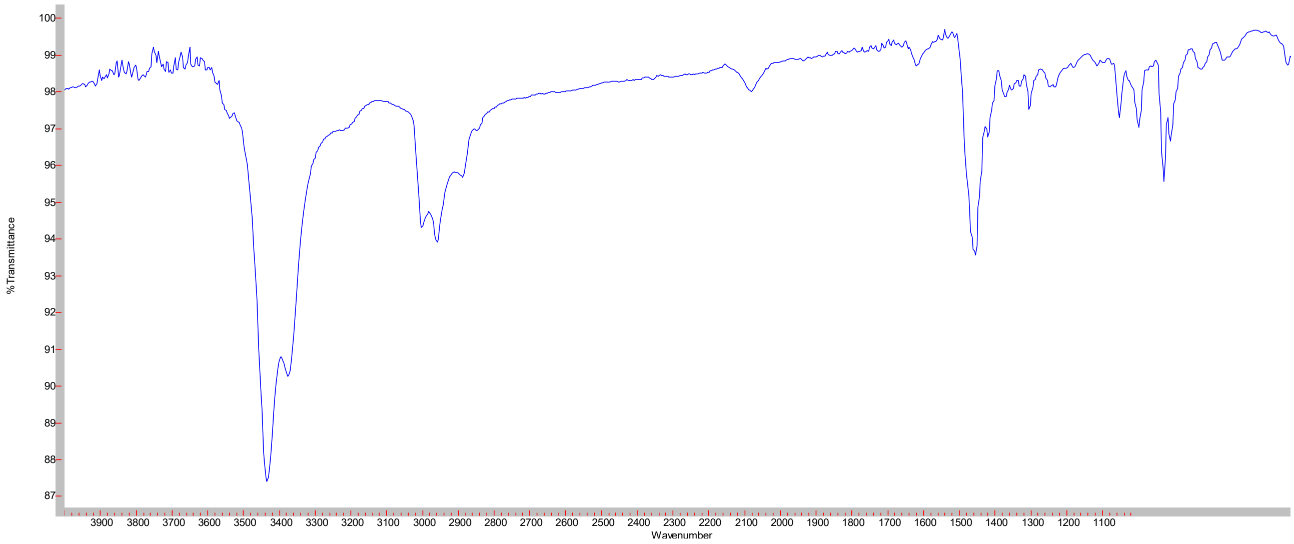

Figure S26. ATR-FTIR spectrum of $[C_3Mpyrr_2]2Br$ (**1**)

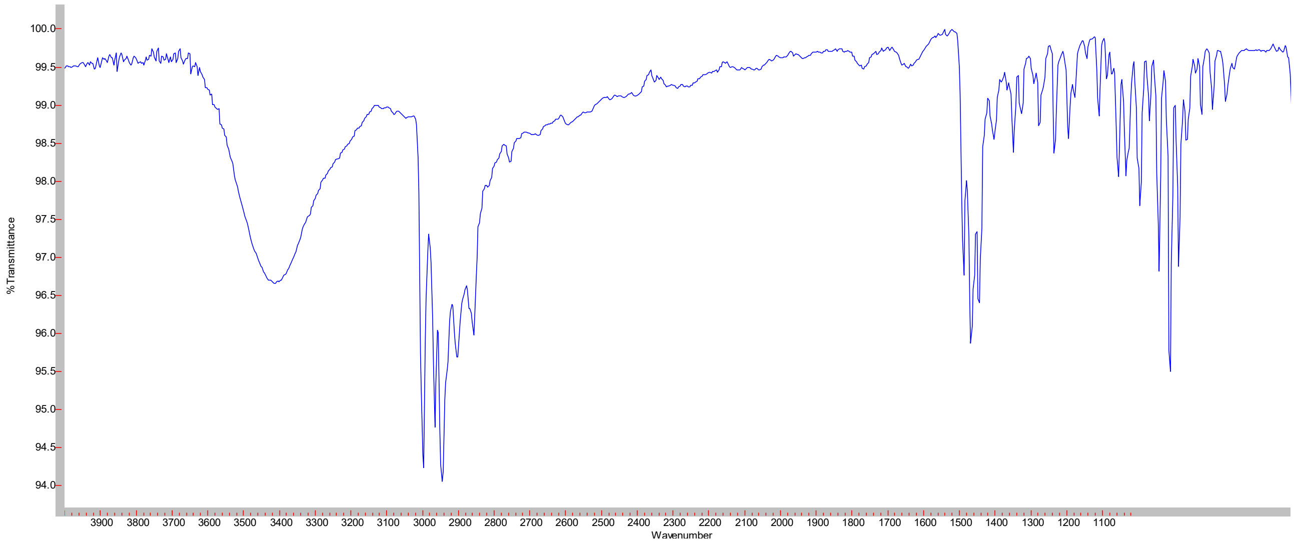

Figure S27. ATR-FTIR spectrum of $[C_3Mpip_2]2Br$ (**2**)

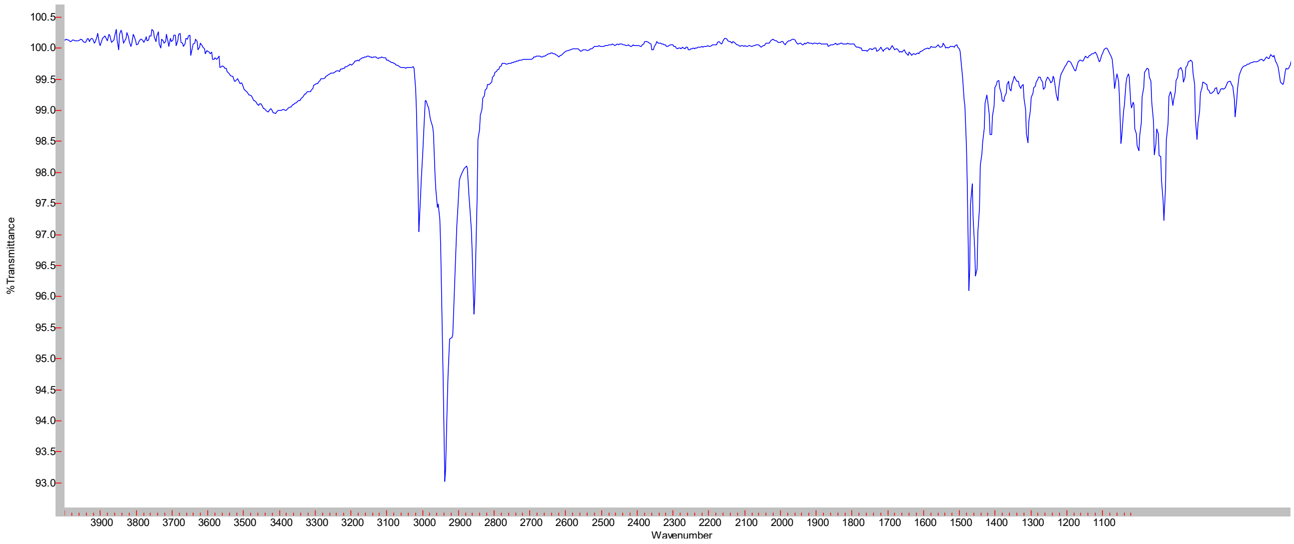

Figure S28. ATR-FTIR spectrum of [C_8Mpyrr$_2$]2Br (**3**)

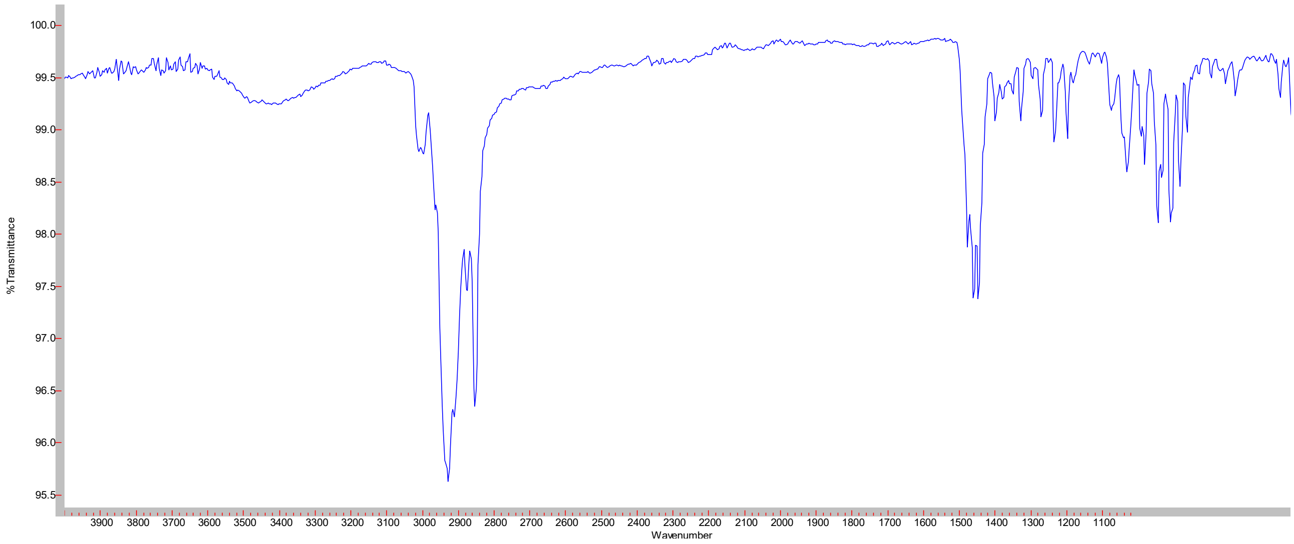

Figure S29. ATR-FTIR spectrum of [C_8Mpip$_2$]2Br (**4**)

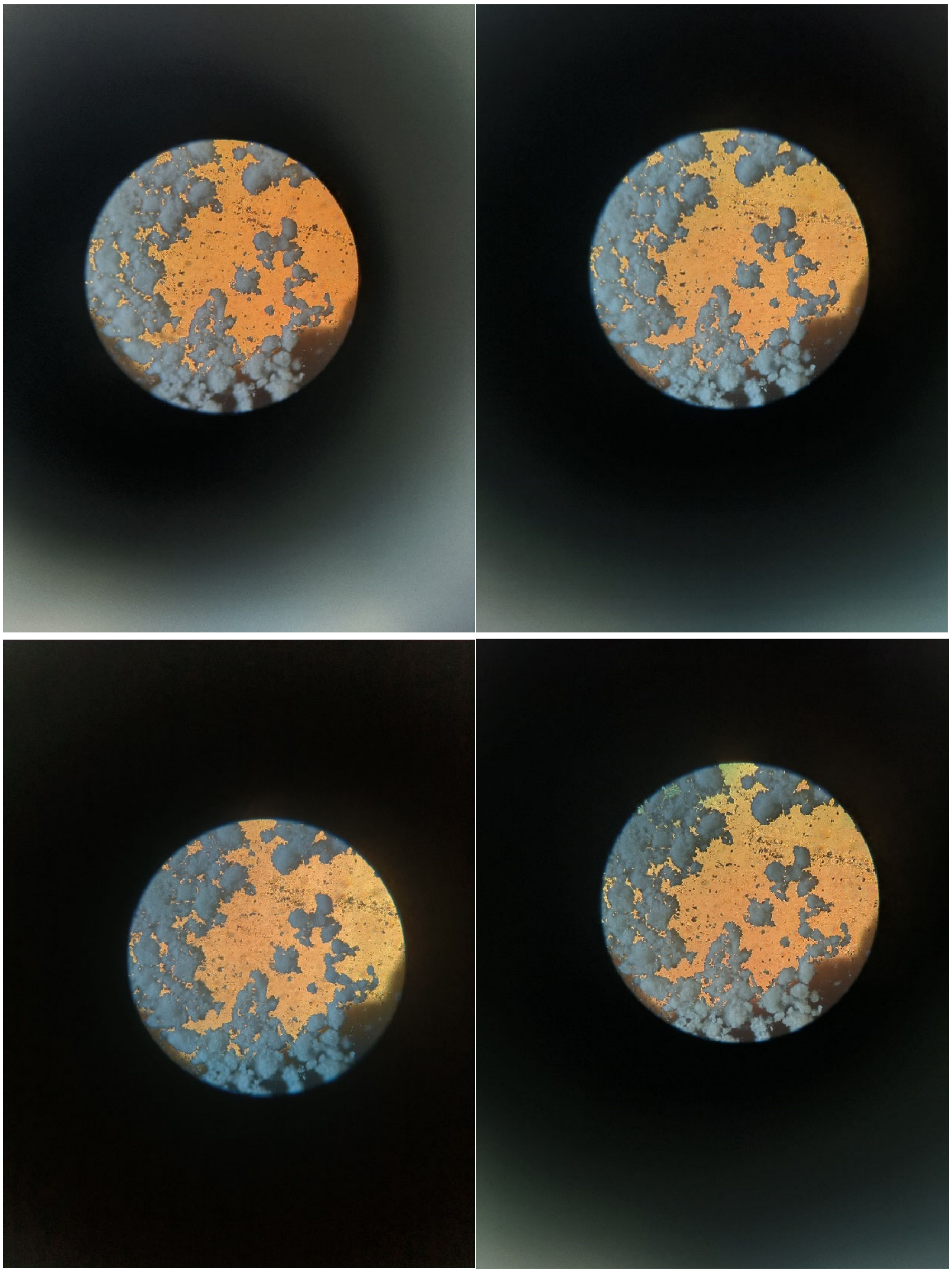

Figure S30. Images of Kofler determination of melting point of [C_3Mpip_2]2Br at 80 °C (upper left), 140 °C (upper right), 180 °C (lower left) and 205 °C (lower right).
